# Simultaneous Production of Aromatics and CO_x_-Free Hydrogen via Methane Dehydroaromatization in Membrane Reactors: A Simulation Study

**DOI:** 10.3390/membranes12121175

**Published:** 2022-11-22

**Authors:** Feng Ye, Shuanshi Fan, Wenjun Li, Yanhong Wang, Xuemei Lang, Jianli Zhang, Jing Li, Gang Li

**Affiliations:** 1 School of Chemistry and Chemical Engineering, South China University of Technology, Guangzhou 510640, China; 2 Beijing Institute of Spacecraft System Engineering, Beijing 100086, China; 3 State Key Laboratory of High-Efficiency Utilization of Coal and Green Chemical Engineering, College of Chemistry and Chemical Engineering, Ningxia University, Yinchuan 750021, China; 4 South China University of Technology-Zhuhai Institute of Modern Industrial Innovation, Zhuhai 519175, China

**Keywords:** methane dehydroaromatization, membrane reactor, CO_x_-free hydrogen, aromatics, hydrogen separation

## Abstract

As an alternative route for aromatics and hydrogen production, methane dehydroaromatization (MDA) is of significant academic and industrial interest due to the abundance of natural gas resources and the intensive demand for aromatics and CO_x_-free hydrogen. In the present work, a simulation study on MDA in membrane reactors (MRs) was performed with the aim of co-producing aromatics and CO_x_-free hydrogen with a highly improved efficiency. The effects of various parameters, including catalytic activity, membrane flux and selectivity, as well as the operating conditions on the MR performance were discussed with respect to methane conversion, hydrogen yield, and hydrogen purity. The results show that catalytic activity and membrane flux and selectivity have significant impacts on CH_4_ conversion and H_2_ yield, whereas H_2_ purity is mainly dominated by membrane selectivity. A highly improved MDA is confirmed to be feasible at a relatively low temperature and a high feed pressure because of the hydrogen extraction effect. To further improve MDA in MRs by intensifying H_2_ extraction, a simple configuration combining a fixed-bed reactor (FBR) and an MR together is proposed for MDA, which demonstrates good potential for the high-efficiency co-production of aromatics and CO_x_-free hydrogen.

## 1. Introduction

Natural gas is the most abundant and cleanest energy carrier among fossil resources; thus, the conversion of methane instead of petroleum and coal into liquid fuels and high-value-added chemicals has been highly desirable in the chemical industry [1,2,3,4,5,6], particularly with the recent boom in the exploitation of unconventional shale gas [7,8,9,10,11] and natural gas hydrate [12,13] resources and with increasing concerns over environmental issues worldwide. Currently, the catalytic conversion of methane to important products, for instance, methanol [14] and olefins [15], is technologically dominated by an indirect route, which involves multistep reactions, and the highly energy-intensive intermediate step for syngas production, either by reforming or by partial oxidation, is generally inevitable, which results in a complicated process with a high production cost and a poor atom economy [16]. Therefore, direct conversion of methane to liquid fuels and chemicals without the syngas production intermediate step is of significant importance for practical applications.

In particular, a direct route, i.e., methane dehydroaromatization (MDA) ($CH_{4}⇌\frac{1}{6}C_{6}H_{6}+\frac{3}{2}H_{2}$) under nonoxidative conditions, has gained significant interest for methane utilization after a pioneering study by Wang et al. [17]. Compared with the conventional aromatic production process using petroleum as the feedstock, the MDA process has been widely accepted as an attractive alternative to produce aromatics with a better sustainability. On the other hand, it is noteworthy that the MDA process can simultaneously generate substantial CO_x_-free hydrogen, which can be directly used as the fuel for polymer electrolyte membrane fuel cells (PEMFCs) with neither the removal of CO, which is a poison to the Pt catalyst, nor the emission of CO_2_, which is mainly responsible for the greenhouse effect. Therefore, CO_x_-free hydrogen produced from MDA demonstrates significant advantages against the conventional methane steam reforming process, i.e., the main route for current hydrogen production with an intensive energy consumption and massive emissions [18,19], in PEMFC applications. Currently, one of the major obstacles to the industrial implementation of the MDA process is its relatively low conversion because MDA is significantly limited by thermodynamic equilibrium. To achieve an acceptable conversion, MDA must be conducted at very high temperatures (≥700 °C) [20,21,22,23,24]; unfortunately, this results in very rapid catalyst deactivation due to the serious coking effect. Thus far, how to simultaneously maintain a high conversion and high catalytic stability for MDA remains a great challenge for industrial applications.

Membrane reactors (MRs), which integrate both separation and catalytic processes into a compact single unit, are promising for enhancing the conversion and/or enabling a lower reaction temperature for thermodynamically limited endothermic reactions because of the equilibrium shift effect. Considering the above advantages against conventional fixed-bed reactors (FBRs), MRs are extremely attractive for high-temperature MDA reactions, and various types of MRs have been successfully developed in an attempt to improve MDA performance [25,26,27,28,29,30,31,32,33]. Among them, most studies focus on Pd and its alloy MRs for MDA application because of its exclusive permeation for hydrogen; however, these Pd-based membranes are very expensive and prone to degradation at high temperatures [26]. Although highly hydrogen-permselective dense ceramic membranes with an excellent stability have been examined as an alternative for MDA application [30,31], the flux of these membranes is too low. On the other hand, in addition to hydrogen-permselective MRs, dense oxygen-permeable ceramic MRs, which allow for finely tuned oxygen distribution in the feed, were also employed for MDA [32,33], and an improved catalytic performance was observed due to the favorable thermodynamics of MDA under oxidative conditions. However, the addition of oxygen to the feed using oxygen-permeable ceramic membranes should be controlled very carefully; otherwise, overoxidation is prone to occur, which results in an extremely low selectivity for MDA. Furthermore, because the generation of CO under oxidative conditions is inevitable, hydrogen produced from oxygen-permeable MRs must be subjected to further purification before use in PEMFCs, which greatly impairs the economic and technical feasibility of the produced hydrogen for PEMFC applications because the separation of CO from hydrogen to an extremely low concentration is both costly and challenging.

Considering the high flux and high selectivity, as well as the excellent thermal stability, porous inorganic membranes, such as zeolite and amorphous silica, are good candidates for MDA applications. Unfortunately, to the best of our knowledge, no porous MRs have been reported for MDA, and details regarding how such factors as the catalytic activity, membrane performance and operating conditions affect the MR performance of MDA in terms of CH_4_ conversion, H_2_ yield and H_2_ purity are still not well understood. In this study, a simulation study of MDA in porous hydrogen-permselective MRs is conducted for the simultaneous production of aromatics and CO_x_-free hydrogen, and insights into the above membrane intensification process would offer important inspiration for developing high-performance porous MRs for practical MDA applications.

## 2. Modeling

**Figure 1:**
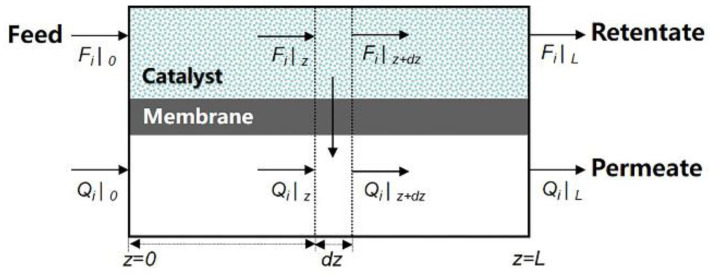
Schematic model for the membrane reactor simulation.

The mathematical model for MDA in concurrent configuration MRs was developed based on the following assumptions: (1) the reactor is isothermal and operates under a steady state, (2) both the feed and permeate streams in the reactor are plug flows and a catalytic reaction only occurs in the feed stream, (3) concentration polarization effects are negligible, and (4) there are no pressure drops in the MR. A schematic model for the simulation study of MRs is shown in Figure 1.

The molar flow rate of component *i* in the MR can be expressed as follows [34,35]:

Feed side

(1) $$\frac{dF_{i}}{dz}=v_{i}Rw_{cat}-sP_{i}(x_{i}p_{h}-y_{i}p_{l})$$

Permeate side

(2) $$\frac{dQ_{i}}{dz}=sP_{i}(x_{i}p_{h}-y_{i}p_{l})$$

where *F_i_* and *Q_i_* are molar flow rates of component *i* in the feed and permeate streams, and *x_i_* and *y_i_* are denoted as their corresponding molar fractions, respectively, *p_h_* and *p_l_* are the pressures of the feed and permeate streams, respectively, *z* is the axial position along the membrane, *v_i_* is the stoichiometric number of component *i*, *P_i_* indicates the permeance of component *i* through the membrane, *w_cat_* and *s* indicate the catalyst weight and membrane area per membrane unit length, respectively, and *R* is the reaction rate of MDA based on the following global reaction equation:

(3) $$CH_{4}⇌\frac{1}{6}C_{6}H_{6}+\frac{3}{2}H_{2}$$

It is believed that a highly stable and selective catalyst for MDA can be expected by carefully tuning the properties of the support and metal species [36,37,38]. Therefore, side reactions in MDA are not considered in the present modeling for simplification in order to evaluate the potential of benzene production in MRs. With the assistance of the dimensionless parameters of the Damköhler number (*Da*), permeation number (*θ*), reaction rate (R^*^), pressure (*p_r_*), permeance ($\alpha_{H_{2}/i}$), and axial position (ζ) defined in Equations (4)–(9):

(4) $$Da=R^{\max}W_{cat}/F_{CH_{4},0}$$

(5) $$\theta =P_{H_{2}}sLp_{h}/F_{CH_{4},0}$$

(6) $$R^{*}=R/R^{\max}$$

(7) $$p_{r}=p_{l}/p_{h}$$

(8) $$\alpha_{H_{2}/i}=P_{H_{2}}/P_{i}$$

(9) ζ=z/L

Equations (1) and (2) can be further expressed as dimensionless forms, as shown in Equations (10) and (11), respectively.

Feed side

(10) $$\frac{df_{i}}{d\zeta }=v_{i}R^{*}Da-\frac{\theta \left(x_{i}-y_{i}p_{r}\right)}{\alpha_{H_{2}/i}}$$

Permeate side

(11) $$\frac{dq_{i}}{d\zeta }=\frac{\theta \left(x_{i}-y_{i}p_{r}\right)}{\alpha_{H_{2}/i}}$$

where *f_i_* and *q_i_* are the dimensionless flow rates normalized by the CH_4_ feed flow rate for component *i* in the feed and permeate streams, respectively, ζ is the axial position of the MR normalized by the membrane length, θ is defined as the permeation number that corresponds to H_2_ flux through the membrane normalized by the CH_4_ feed low rate, $\alpha_{H_{2}/i}$ is the permeance ratio of H_2_ to component *i*, *p_r_* is the pressure ratio of the permeate stream to the feed stream, R^*^ is the ratio of the reaction rate *R* to the maximum reaction rate *R^max^* based on the inlet feed composition, *Da* is the Damköhler number, which is defined as the ratio of the product of the maximum reaction rate *R^max^* and the total catalyst weight of the membrane module *W_cat_* to the CH_4_ feed flow rate, and *Da* indicates the effect of the catalyst and the feed flow rate on the catalytic performance, which can be used as a measure of the catalytic activity. The higher *Da* is, the closer the reaction is to equilibrium.

According to a simple single-site mechanism [39], the global reaction of MDA (Equation (3)) consists of the following series of elementary steps:

(12) $$I.\;CH_{4}+*\overset{K_{1}}{⇌}CH_{4}*$$

(13) $$\mathrm{II}.\;CH_{4}*\overset{K_{2}}{⇌}CH_{2}*+H_{2}$$

(14) $$\mathrm{III}.\;CH_{2}*\overset{K_{3}}{⇌}\frac{1}{2}C_{2}H_{4}+*$$

(15) $$\mathrm{IV}.\;\frac{1}{2}C_{2}H_{4}\overset{K_{4}}{⇌}\frac{1}{6}C_{6}H_{6}+\frac{1}{2}H_{2}$$

where * indicates the active site of the catalyst, and step III is obtained by merging the CH_2_ dimerization and C_2_H_4_ desorption steps in Equations (16) and (17):

(16) $$V.\;CH_{2}*\overset{K_{5}}{⇌}\frac{1}{2}C_{2}H_{4}*$$

(17) $$\mathrm{VI}.\;\frac{1}{2}C_{2}H_{4}*\overset{K_{6}}{⇌}\frac{1}{2}C_{2}H_{4}+*$$

Step II is considered to be the rate-determining step in MDA, yielding a reaction rate expression for the global reaction in Equation (18) [39].

(18) $$R=k_{2}\frac{P_{CH_{4}}-\frac{1}{K_{p}}P_{C_{6}H_{6}}^{1/6}P_{H_{2}}^{3/2}}{1+K_{1}P_{CH_{4}}+\frac{K_{3}}{K_{4}}P_{C_{6}H_{6}}^{1/6}P_{H_{2}}^{1/2}}$$

where

(19) $$K_{4}=\exp\left(-\frac{\Delta G_{4}}{R_{g}T}\right){\left(p^{\Theta }\right)}^{\frac{1}{6}}$$

(20) $$K_{p}=\exp\left(-\frac{\Delta G}{R_{g}T}\right){\left(p^{\Theta }\right)}^{\frac{2}{3}}$$

$p^{\Theta }$ is the standard pressure, $P_{CH_{4}}$, $P_{C_{6}H_{6}}$ and $P_{H_{2}}$ are the partial pressures of CH_4_, C_6_H_6_, and H_2_, respectively, Δ*G* and Δ*G*_4_ are the Gibbs free energies of the global (Equation (1)) and step IV (Equation (15)) reactions, respectively, *k*_2_ is the reaction rate constant of step II, and *K*_1_, *K*_3_, *K*_4_ and *K_p_* are equilibrium constants for steps I, III, IV and the global reaction, respectively. For a 0.5%Ru-3%Mo/ZSM-5 catalyst, the reaction rate and equilibrium constants (*K*_1_, *K*_3_, *k*_2_) are shown in Table 1 according to Iliuta et al. [39], which were adopted in the present work for the simulation study.

The simulation study of MDA in concurrent MRs was performed at temperatures of 873–973 K, with porous H_2_-permselective membranes, showing H_2_/CH_4_ selectivities in the range of 10–∞, under feed and permeate pressures of 100–1000 and 5 kPa, respectively. Because both CH_4_ and C_6_H_6_ are much larger than H_2_ in terms of molecular size, they are assumed to permeate through the defects of H_2_-permselective membranes based on a Knudsen diffusion mechanism; therefore, the selectivity of CH_4_ to C_6_H_6_ in the MR is fixed to the Knudsen selectivity in the simulation. Because benzene is a condensable product that is easily separated and collected on both retentate and permeate sides of the MR, the yield of benzene in the MR is defined based on the product obtained in both retentate and permeate streams, and the effect of the presence of benzene on the final H_2_ purity can be negligible. On the other hand, both H_2_ yield and H_2_ purity is defined based on only the permeate side, because purified H_2_ is highly desirable. Therefore, the CH_4_ conversion ($X_{CH_{4}}$), benzene yield ($Y_{C_{6}H_{6}}$), H_2_ yield ($Y_{H_{2}}$), and H_2_ purity ($C_{H_{2}}$) obtained in the MRs are defined as follows:

(21) $$X_{CH_{4}}=\frac{F_{CH_{4},0}-F_{CH_{4},L}-Q_{CH_{4},L}}{F_{CH_{4},0}}$$

(22) $$Y_{C_{6}H_{6}}=\frac{6F_{C_{6}H_{6},L}+6Q_{C_{6}H_{6},L}}{F_{CH_{4},0}}$$

(23) $$Y_{H_{2}}=\frac{2Q_{H_{2},L}}{3F_{CH_{4},0}}$$

(24) $$C_{H_{2}}=\frac{Q_{H_{2},L}}{Q_{CH_{4,}L}+Q_{H_{2},L}}$$

For FBRs, the CH_4_ conversion, benzene yield, H_2_ yield and H_2_ purity are defined in Equations (25)–(28):

(25) $$X_{CH_{4}}=\frac{F_{CH_{4},0}-F_{CH_{4},L}}{F_{CH_{4},0}}$$

(26) $$Y_{C_{6}H_{6}}=\frac{6F_{C_{6}H_{6},L}}{F_{CH_{4},0}}$$

(27) $$Y_{H_{2}}=\frac{2F_{H_{2},L}}{3F_{CH_{4},0}}$$

(28) $$C_{H_{2}}=\frac{F_{H_{2},L}}{F_{CH_{4,}L}+F_{H_{2},L}}$$

## 3. Results and Discussion

### 3.1. Thermodynamic Analysis and Model Validation

**Figure 2:**
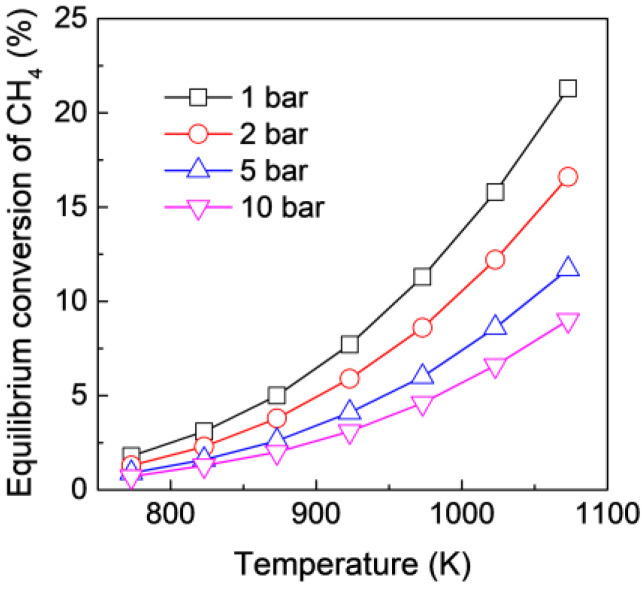
Equilibrium conversion of CH_4_ in MDA under different temperatures and pressures.

Figure 2 shows the equilibrium conversion of CH_4_ in MDA under different temperatures and feed pressures. The equilibrium conversion of CH_4_ is quite low, particularly under a low temperature and a high pressure. The above calculation confirms that MDA is significantly thermodynamically unfavorable. Although CH_4_ conversion in MDA can be improved by increasing the reaction temperature, catalyst deactivation is reportedly even faster due to the more serious coking effect at a higher temperature [40]. Therefore, to strike a balance between CH_4_ conversion and catalytic stability, MDA is generally conducted at 700 °C and at atmospheric pressure [21,22,23], which corresponds to an equilibrium conversion of approximately 12% due to the significant thermodynamic limitation. The above results demonstrate that there is significant potential to use H_2_-permselective MRs for highly enhanced MDA because the equilibrium of MDA can be efficiently shifted to the product side after selective H_2_ extraction.

**Figure 3:**
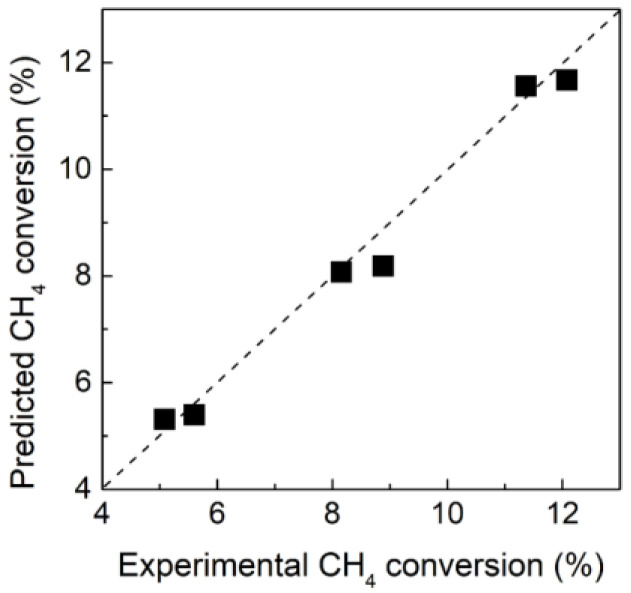
Comparison of experimentally obtained and theoretically predicted CH_4_ conversions in the MR without H_2_ extraction.

To validate the proposed model for the simulation study of MDA in MRs, we first calculated the CH_4_ conversion based on the present model and previously reported experimental parameters [39] using the same cylindrical reactor without H_2_ extraction under various operating conditions. As shown in Figure 3, both the theoretically predicted and experimentally obtained CH_4_ conversions showed an excellent agreement, which verifies the feasibility of the proposed model for the simulation study of MDA. Unfortunately, further verification cannot be conducted by applying H_2_ extraction to MRs because Pd-based membranes are prone to poison in the presence of hydrocarbons at high temperatures, and the actual H_2_ permeation performance of the membrane reactor, which is reportedly significantly different compared with that obtained during the H_2_ permeation test [28], is currently not yet available during MDA.

### 3.2. Effect of Catalysts on the MR Performance

**Figure 4:**
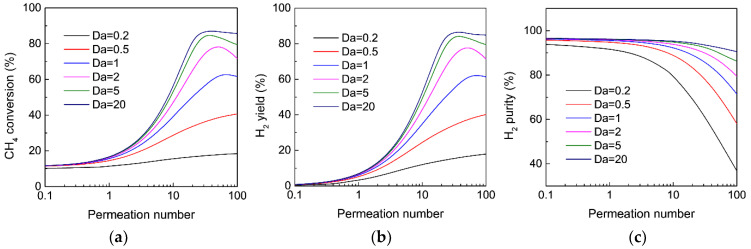
Effect of the Damköhler number on (**a**) CH_4_ conversion, (**b**) H_2_ yield, and (**c**) H_2_ purity as a function of permeation number for MDA in the MR. (Simulation conditions: *T* = 973 K, $\alpha_{H_{2}/CH_{4}}$ = 200, $\alpha_{CH_{4}/C_{6}H_{6}}$ = Knudsen selectivity, *p_h_* = 1 bar, *p_l_* = 0.05 bar).

Figure 4 shows the effect of the Damköhler number on CH_4_ conversion, H_2_ yield, and H_2_ purity as a function of the permeation number for MDA in the MR. When the catalytic activity of the MR is relatively low, for instance, *Da* = 0.2 and 0.5, both CH_4_ conversion and H_2_ yield obtained in the MR gradually increased with an increasing permeation number, which could be ascribed to the improved equilibrium shift effect, since H_2_ extraction from the MR is enhanced. For MR with a relatively active catalyst (*Da* ≥ 1), the improvement in the CH_4_ conversion and H_2_ yield is more remarkable due to the enhanced driving force for H_2_ extraction. However, both the CH_4_ conversion and H_2_ yield show a maximum as the permeation number increases, which could be mainly attributed to the adverse effect of CH_4_ permeation through the membrane because the amount of CH_4_ permeation through the membrane becomes remarkable at high *Da* values and permeation numbers. Similar trends were also previously observed for various dehydrogenation reactions in H_2_-permselective MRs [34,35]. On the other hand, the H_2_ purity in the permeate stream always decreases with an increasing permeation number, regardless of the values of *Da*, which mainly results from the enhanced CH_4_ permeation through the membrane at a high permeation number. However, for a given permeation number, H_2_ purity increases as *Da* increases because CH_4_ conversion is improved and the permeation of CH_4_ through the membrane becomes slower. The above results demonstrate that an active catalyst is necessary for the effective enhancement of MDA in an MR with respect to CH_4_ conversion, H_2_ yield and H_2_ purity.

### 3.3. Effect of Membranes on the MR Performance

**Figure 5:**
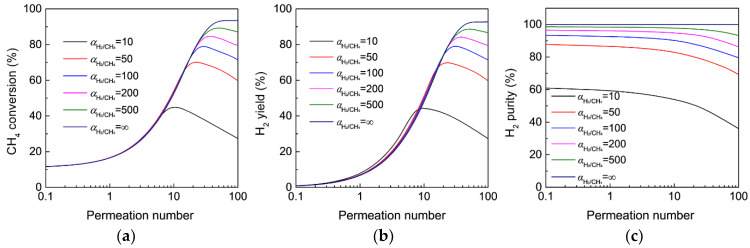
Effect of H_2_/CH_4_ selectivity on (**a**) CH_4_ conversion, (**b**) H_2_ yield, and (**c**) H_2_ purity as a function of permeation number for MDA in the membrane reactor. (Simulation conditions: *T* = 973 K, *Da* = 5, $\alpha_{CH_{4}/C_{6}H_{6}}$ = Knudsen selectivity, *p_h_* = 1 bar, *p_l_* = 0.05 bar).

Figure 5 shows the effect of H_2_/CH_4_ selectivity of the membrane on CH_4_ conversion, H_2_ yield, and H_2_ purity as a function of permeation number for MDA in the MR. As the permeation number increases from 0.1 to 100, both the CH_4_ conversion and H_2_ yield in porous MRs with a given H_2_/CH_4_ selectivity initially increase and then subsequently decrease, resulting in a maximum. This is because the equilibrium shift effect after H_2_ extraction that contributes to the improved CH_4_ conversion and yield is dominative at a relatively low permeation number, whereas the effect of CH_4_ permeation from the feed to the permeate side that lowers the CH_4_ conversion and yield becomes very important at a high permeation number. The maximal CH_4_ conversion and H_2_ yield are effectively enhanced as the H_2_/CH_4_ selectivity of the MR is increased from 10 to 500 because the leakage of CH_4_ from the feed to permeate sides can be significantly reduced with a highly H_2_-selective membrane. Note that the MR shows a low degree of improvement in CH_4_ conversion and H_2_ yield at a low permeation number, even though the membrane selectivity is extremely high. This is because only a very limited amount of H_2_ is extracted from the reactor at a relatively low permeation number. Therefore, a remarkable improvement is achieved only for MR with both an acceptable H_2_ flux and selectivity.

Considering the excellent H_2_ separation performance of Pd-based membranes, MDA in MRs should be effectively improved after H_2_ extraction according to the present and previously reported theoretical simulations [41,42]. However, experimental investigations [25,26,28] have demonstrated that Pd-based MRs show only a very limited enhancement for MDA compared with FBRs under the same operating conditions. We believe that the remarkable difference between the theoretical prediction and the experimental results is most likely attributed to the significantly decreased H_2_ permeation flux of Pd-based membranes during MDA because Pd-based membranes are reportedly poisoned in the presence of hydrocarbons at high temperatures [43], which significantly hinder H_2_ permeation by blocking Pd sites on the membrane surface from adsorbing and dissociating H_2_ molecules. Recently, Natesakhawat et al. [28] compared the H_2_ permeation performance of a tubular Pd membrane in 10% H_2_/N_2_ and 10% H_2_/CH_4_ mixtures at 700 °C to study the effect of CH_4_ on the Pd membrane performance. The Pd membrane in a later system lost up to 75% of its H_2_ permeability, which confirms the occurrence of serious CH_4_ poisoning for the Pd membrane. Moreover, the reduction of H_2_ permeability of Pd membranes was reportedly even much more noticeable during MDA [28]. The above simulation results confirm that the poor catalytic performance of Pd-based MRs in experimental investigations most likely results from the severe degradation of H_2_ permeation flux during MDA.

Importantly, it is worth noting that the porous MR with a H_2_/CH_4_ selectivity of 200 shows almost the same MDA performance in terms of the CH_4_ conversion and H_2_ yield compared with those of the dense Pd-based MR with an infinite H_2_/CH_4_ selectivity under the same permeation number ranging from 0.1 to 30, and both the maximal CH_4_ conversion and H_2_ yield exceed 80%, which demonstrates a significant potential for highly enhanced MDA in the porous MRs. Although dense Pd membranes show an exclusive permeation for H_2_, taking their relatively lower flux during MDA and the high cost into consideration, porous MRs with an acceptable cost and H_2_/CH_4_ selectivity (>200) but a much higher H_2_ permeance (>10^−6^ mol Pa^−1^ m^−2^ s^−1^) free of CH_4_ poisoning, such as amorphous silica membranes [44,45,46], would be preferable for MDA in practical applications in terms of the CH_4_ conversion and H_2_ yield.

The H_2_ purity obtained in the MR can be significantly enhanced as the membrane selectivity increases, and the effect of the permeation number seems to have much less of an impact on the H_2_ purity, particularly for the MR with a H_2_ selectivity over 200. The above results demonstrate that both the CH_4_ conversion and H_2_ yield obtained in the MR are greatly affected by both the catalytic activity and membrane performance, while the H_2_ purity is mainly dominated by the membrane selectivity. In this regard, dense Pd membranes with exclusive H_2_ permeation are preferable when the H_2_ purity is a priority for MDA in the MR. Therefore, the selection of membrane materials should be carefully considered according to the objective MR performance in terms of the CH_4_ conversion, H_2_ yield, and H_2_ purity.

### 3.4. Effect of Operating Conditions on the MR Performance

**Figure 6:**
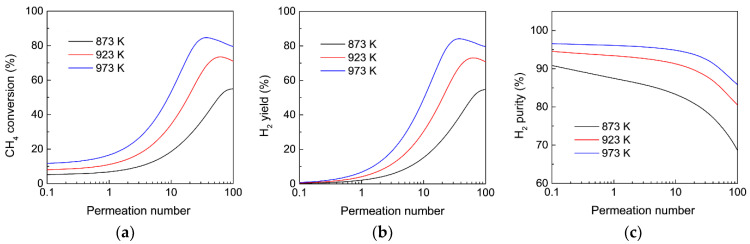
Effect of the reaction temperature on (**a**) CH_4_ conversion, (**b**) H_2_ yield, and (**c**) H_2_ purity as a function of the permeation number for MDA in the membrane reactor. (Simulation conditions: *Da* = 5, $\alpha_{H_{2}/CH_{4}}$ = 200, $\alpha_{CH_{4}/C_{6}H_{6}}$ = Knudsen selectivity, *p_h_* = 1 bar, *p_l_* = 0.05 bar).

Thermodynamically, the coking reaction is much more favorable than MDA at a high temperature and leads to catalyst deactivation during MDA. To reduce the effect of coking on the catalytic stability, it is preferable to perform MDA under a relatively low temperature, which would impair the CH_4_ conversion for MDA in the conventional FBR. However, MR allows for the possibility that MDA is conducted at a lower temperature, whereas the CH_4_ conversion remains high because of the equilibrium shift effect after H_2_ extraction. Figure 6 shows the effect of the reaction temperature on the CH_4_ conversion, H_2_ yield, and H_2_ purity as a function of the permeation number for MDA in the MR. As expected, for a given permeation number, although CH_4_ conversion, H_2_ yield, and H_2_ purity all decrease with a decreasing reaction temperature because MDA is thermodynamically unfavorable at a low temperature, the MR performance greatly surpasses that obtained in the conventional FBR under the same reaction temperature, and the improvement is still extremely remarkable, even under 873 K if the MR is highly permeable. The above results demonstrate the significant importance of intensifying the MR performance for MDA by improving H_2_ permeance via reducing the membrane thickness. This is particularly important for MDA operated under a lower reaction temperature because the H_2_ partial pressure difference across the membrane is lower for H_2_ permeation. Recent progress has demonstrated that emerging two-dimensional-material membranes [47] constructed by 2D nanosheets with few atomic thickness layers show both a high flux and high selectivity, which would be a future candidate for the development of high-performance MRs for MDA.

**Figure 7:**
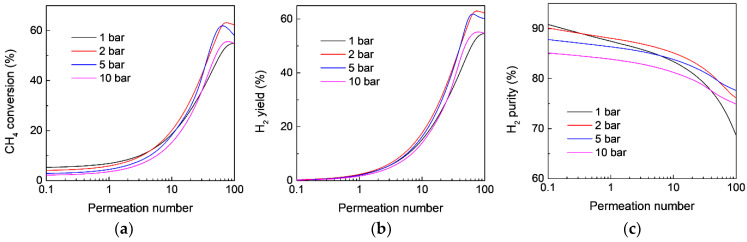
Effect of the feed pressure on (**a**) CH_4_ conversion, (**b**) H_2_ yield, and (**c**) H_2_ purity as a function of the permeation number for MDA in the membrane reactor. (Simulation conditions: *T* = 873 K, *Da* = 5, $\alpha_{H_{2}/CH_{4}}$ = 200, $\alpha_{CH_{4}/C_{6}H_{6}}$ = Knudsen selectivity, *p_l_* = 0.05 bar).

**Figure 8:**
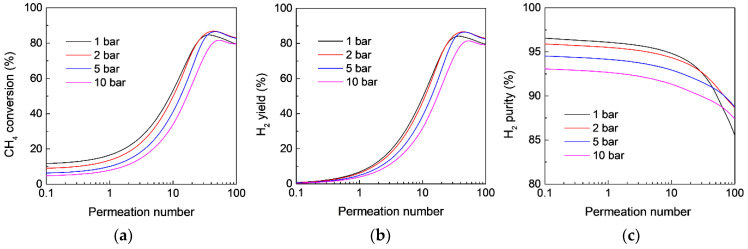
Effect of CH_4_ feed pressure on (**a**) CH_4_ conversion, (**b**) H_2_ yield, and (**c**) H_2_ purity as a function of the permeation number for MDA in the membrane reactor. (Simulation conditions: *T* = 973 K, *Da* = 5; $\alpha_{H_{2}/CH_{4}}$ = 200, $\alpha_{CH_{4}/C_{6}H_{6}}$ = Knudsen selectivity, *p_l_* = 0.05 bar).

On the other hand, although a high feed pressure is not favorable for MDA in an FBR according to the equilibrium shift by Le Chatelier’s principle, as also confirmed in the aforementioned calculation (Figure 2), the effect of the feed pressure on MDA in the MR is more complicated because a high pressure also favors H_2_ permeation through the membrane, which is believed to boost MDA in MRs from the viewpoint of an equilibrium shift due to the enhancing H_2_ extraction. Therefore, whether a high feed pressure benefits MDA in MRs depends on the equilibrium shift effect caused by both H_2_ extraction and the feed pressure. However, how the feed pressure affects MDA in MRs remains unclear. Figure 7 and Figure 8 show the effect of the feed pressure on CH_4_ conversion, H_2_ yield, and H_2_ purity as a function of permeation number for MDA in the MR at 873 and 973 K, respectively. For the same permeation number with a relatively low value, the MR shows a decreased CH_4_ conversion with an increasing feed pressure, regardless of the reaction temperature, because the final CH_4_ conversion is largely affected by the equilibrium shift effect by high feed pressures. However, when the permeation number is larger than 10, compared with MDA in the MR under atmospheric pressure, the MR shows an enhanced CH_4_ conversion along with an improved H_2_ yield and purity in a pressurized system, although the high pressure is unfavorable for MDA in FBRs, which could be ascribed to the significant contribution of the equilibrium shift effect by enhanced H_2_ extraction at high feed pressures. It should be noted that the MR performance could again deteriorate at much higher pressures because the effect of feed pressure on the MR performance becomes more remarkable. For instance, the CH_4_ conversion at a feed pressure of 10 bar is lower than that obtained at atmospheric pressure at 873 K, even though the MR is highly permeable (Figure 8). Therefore, there is an optimal feed pressure to maximize the MDA performance in MRs, and the value highly depends on the specific reaction conditions used, such as temperature and catalyst.

### 3.5. Integration of an FBR and an MR for Enhanced MDA

**Figure 9:**
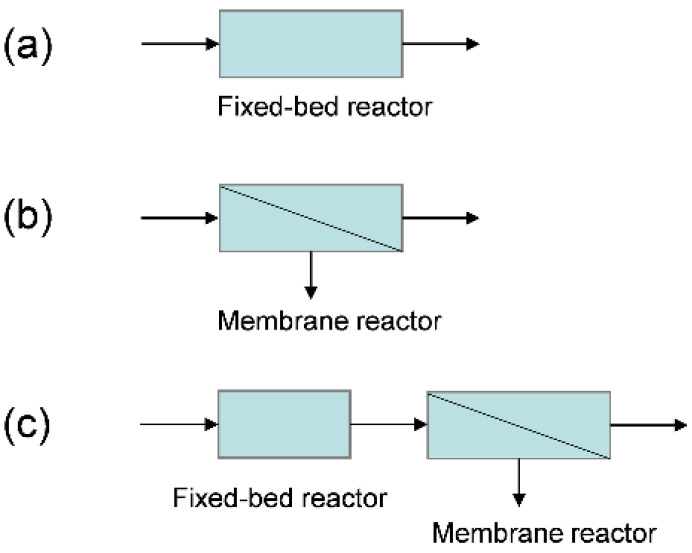
Different reactor configurations used for MDA. (**a**) Fixed-bed reactor; (**b**) membrane reactor; (**c**) integration of a fixed-bed reactor and a membrane reactor.

The enhancement of the MR performance is significantly affected by the amount of extracted H_2_ during the reaction, and thus a high membrane packing density is highly desirable for practical MDA applications. Hollow fiber MRs are generally preferable when taking the very high packing density into account [48]. However, one of the major obstacles to the practical use of hollow fiber MRs is the relatively low catalyst loading capacity, which generally results in a poor performance of MRs due to the relatively low catalytic activity, as confirmed in Figure 4. This is because the H_2_ partial pressure across the membrane, which is the driving force for H_2_ permeation, is too low to extract sufficient H_2_ from the MR. Consequently, H_2_ permeation in hollow fiber MRs should be further intensified to more efficiently promote the MDA performance. To achieve a higher MDA performance, a facile configuration that integrates a fixed-bed reactor (FBR) and an MR is suggested for MDA in which the FBR functions as a pre-reactor. For comparison, the performance of MDA in a single conventional FBR and a single MR are studied under identical operating conditions. The schematic reactor configurations are shown in Figure 9.

**Figure 10:**
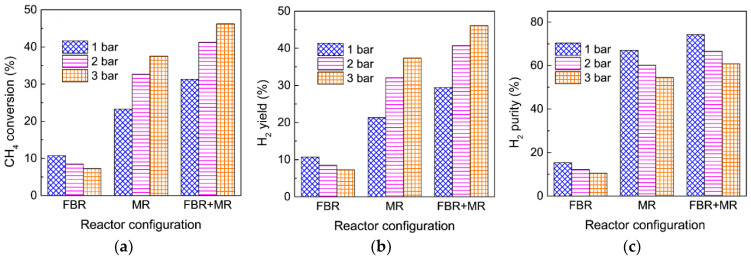
Effect of reactor configurations on (**a**) CH_4_ conversion, (**b**) H_2_ yield, and (**c**) H_2_ purity for MDA in the membrane reactor with different CH_4_ feed pressures. (Simulation conditions: Temperature = 973 K, catalyst amount = 0.15 g, CH_4_ feed = 6.59 × 10^−3^ mol h^−1^, H_2_ permeance = 72 mol m^−2^ h^−1^ bar^−1^, membrane length = 0.4 m, membrane diameter = 2.5 × 10^−3^ m, $\alpha_{H_{2}/CH_{4}}$ = 200, $\alpha_{CH_{4}/C_{6}H_{6}}$ = Knudsen selectivity, *p_l_* = 0.05 bar, and *p_h_* = 1, 2 and 3 bar).

**Figure 11:**
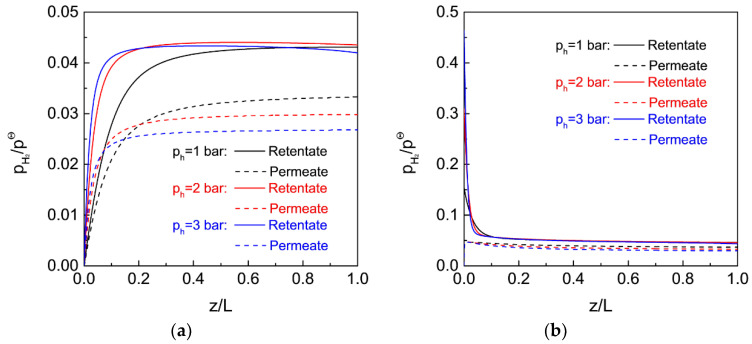
Axial profiles of H_2_ partial pressure normalized by the standard pressure for MDA in the membrane reactor under different feed pressures (**a**) with and (**b**) without the assistance of a pre-reactor. (Simulation conditions: Temperature = 973 K, catalyst amount = 0.15 g, CH_4_ feed = 6.59 × 10^−3^ mol h^−1^, H_2_ permeance = 72 mol m^−2^ h^−1^ bar^−1^, membrane length = 0.4 m, membrane diameter = 2.5 × 10^−3^ m, $\alpha_{H_{2}/CH_{4}}$ = 200, $\alpha_{CH_{4}/C_{6}H_{6}}$ = Knudsen selectivity, *p_l_* = 0.05 bar, and *p_h_* = 1, 2 and 3 bar).

Figure 10 shows the effect of reactor configurations on the CH_4_ conversion, H_2_ yield, and H_2_ purity for MDA in the MR under different feed pressures. For a comparison, catalyst loading in the FBR (Figure 9a) and MRs (Figure 9b,c) are fixed at the same amount, while the CH_4_ conversion in the FBR (Figure 9c) is assumed to achieve equilibrium under atmospheric pressure because a high catalyst loading in the FBR is practically feasible. For the single conventional FBR (Figure 9a), the CH_4_ conversion, H_2_ yield and H_2_ purity are 10.7, 10.7 and 10.5%, respectively, at a feed pressure of 1 bar, and all of these values decrease with an increasing feed pressure, which can be ascribed to the equilibrium shift of MDA to the backward side at high feed pressures. However, the MDA performance is much enhanced in the single MR, particularly under a high feed pressure, although a pressurized system is unfavorable for MDA in an FBR. As the feed pressure increases from 1 to 2 and 3 bar, the CH_4_ conversion gradually increases from 23.3 to 32.7 and 37.6%, with the H_2_ yield increasing from 21.3 to 32.1 and 37.3%, and the H_2_ purity decreasing from 74.3 to 66.6 and 60.1%, respectively. This remarkable improvement in the MDA can be ascribed to the equilibrium shift effect of the MR after H_2_ extraction, where a higher feed pressure effectively promotes H_2_ extraction due to the higher H_2_ partial pressure difference across the membrane, as evidenced by the axial profiles of the normalized H_2_ partial pressure in the MR under different feed pressures in Figure 11a. The above result demonstrates that the MR performance under present pressurized conditions is largely affected by the equilibrium shift effect caused by H_2_ extraction, rather than by a high pressure. When an FBR is coupled with the MR (Figure 9c), after completion of the reaction in the FBR, the H_2_ partial pressure is quite high at the inlet of the MR, which would facilitate H_2_ extraction during the subsequent MR due to the increased driving force for H_2_ permeation. As expected, CH_4_ conversion in the MR is further improved to 31.3% at a feed pressure of 1 bar with the assistance of an FBR, and a higher feed pressure in the MR even improves the CH_4_ conversion to a higher degree (41.2 and 46.2% at 2 and 3 bar, respectively). The corresponding H_2_ yield also increases from 29.4 to 40.7 and 46.1%, whereas the H_2_ purity decreases from 74.3 to 66.6 and 60.1 at 1, 2 and 3 bar, respectively. Previously, an improved conversion was also observed for methylcyclohexane dehydrogenation in an MR when a FBR was coupled [49]. With the assistance of FBR as a pre-reactor, the exceptional MDA performance in the MR was primarily ascribed to the enhanced H_2_ extraction. As confirmed by the axial profiles of the normalized H_2_ partial pressure for MDA in the MR in Figure 11b, the employment of a pre-reactor indeed increases the H_2_ partial pressure difference across the membrane, particularly near the inlet zone of the MR. This effect is even more effective under pressurized systems, which further promotes the equilibrium shift of MDA to the forward side and results in a much higher CH_4_ conversion and H_2_ yield and purity than those obtained in the single FBR and the single MR. 

Considering the effectively enhanced H_2_ extraction, the proposed simple reactor configuration that combines an FBR and an MR is superior to both the conventional single FBR and the single MR in terms of the CH_4_ conversion, H_2_ yield and H_2_ purity. In addition, it should be noted that MDA is endothermic; therefore, heat transfer to the catalyst bed must be fast enough to maintain a constant temperature in the reactor. The new reactor configuration consisting of two parts allows for sectional heating for MDA, which again benefits MDA for simultaneous large-scale production of aromatics and CO_x_-free H_2_ in industrial applications under isothermal conditions.

## 4. Conclusions

A dimensionless mathematical model was formulated and verified for a simulation study on methane dehydroaromatization in H_2_-permselective MRs for the simultaneous production of aromatics and CO_x_-free H_2_. The simulation results showed that the improvement in the CH_4_ conversion and H_2_ yield in the MR is strongly influenced by the catalytic activity and membrane flux and selectivity, whereas H_2_ purity is mainly determined by the membrane selectivity. Compared with dense Pd-based membranes, porous membranes, such as amorphous silica, with a H_2_/CH_4_ selectivity of several hundred are highly preferable for MDA with respect to the both CH_4_ conversion and H_2_ yield due to the high H_2_ permeance and the absence of CH_4_ poisoning. Operating conditions, including the reaction temperature and pressure, also exert important impacts on the MR performance. A low temperature generally results in a decrease in the MDA performance due to unfavorable thermodynamics, but a significantly higher performance, compared with that of conventional FBRs, can still be achieved in MRs due to the equilibrium shift effect after H_2_ extraction. Therefore, MRs have great potential to lower the reaction temperature and thus reduce the coking on the catalyst for MDA. A pressurized system is confirmed to further improve MDA in the MR under certain conditions due to the enhanced H_2_ extraction, although a pressurized system is unfavorable for MDA in FBRs. Finally, a simple configuration that combines an FBR and an MR is proposed for MDA, which demonstrates a much better performance in terms of the CH_4_ conversion, H_2_ yield, and H_2_ purity compared with both the single FBR and single MR due to the intensified H_2_ extraction. This simple and effective configuration shows a great potential for the simultaneous production of aromatics and CO_x_-free H_2_ with a high efficiency for practical applications.

## Figures and Tables

**Table 1:** Reaction rate and equilibrium constants used in the present simulation.

Temperature (K)	*k*_2_ (mol g_cat_^−1^ h^−1^ bar^−1^)	*K*_1_ (bar^−1^)	*K*_3_ (bar^−1/2^)
873	0.00717	2.877	2.359
898	0.0102	2.197	2.870
923	0.014	1.675	3.020
948	0.019	1.280	3.185
973	0.025	1.029	3.300

## Data Availability

Not applicable.

## Nomenclature

*Da*	Damköhler number, dimensionless
*f*	dimensionless feed-side flow rate, dimensionless
*F*	feed-side molar flow rate, mol h^−1^
*C*	purity of hydrogen, dimensionless
*k*	reaction rate constant, mol g_cat_^−1^ h^−1^ bar^−1^
*k*_2_	reaction rate constant, mol g_cat_^−1^ h^−1^ bar^−1^
*K*_1_	equilibrium constant, bar^−1^
*K*_3_	equilibrium constant, bar^−1/2^
*K*_4_	equilibrium constant, bar^1/6^
*K*_*p*_	equilibrium constant, bar^2/3^
*L*	membrane reactor length, m
*p*	partial pressure, bar
$p^{\Theta }$	standard pressure, bar
*p*_*h*_	feed-side pressure, bar
*p*_*l*_	permeate-side pressure, bar
*p*_*r*_	pressure ratio, dimensionless
*P*	gas permeance, mol m^−2^ h^−1^ bar^−1^
*q*	dimensionless permeate-side flow rate, dimensionless
*Q*	permeate-side molar flow rate, mol h^−1^
*R*	reaction rate, mol g_cat_^−1^ h^−1^
*R*_*g*_	ideal gas constant, J mol^−1^ K^−1^
*R*_*max*_	maximum reaction rate, mol g_cat_^−1^ h^−1^
*R* ^*^	dimensionless reaction rate, dimensionless
*s*	membrane area per unit membrane axial length, m^2^ m^−1^
*T*	absolute temperature, K
*w*_*cat*_	catalyst weight per unit membrane axial length, g_cat_ m^−1^
*W*_*cat*_	catalyst weigh of the membrane module, g
*x*	feed-side mole fraction, dimensionless
*X*	conversion, dimensionless
*y*	permeate-side mole fraction, dimensionless
*Y*	yield, dimensionless
*z*	axial coordinate, m
Greek letters	
*α*	permeance ratio, dimensionless
*θ*	permeation number, dimensionless
*ν*	stoichiometric coefficient, dimensionless
*ζ*	dimensionless axial coordinate, dimensionless
Δ*G*	Gibbs free energy, J mol^−1^
Δ*G*_4_	Gibbs free energy, J mol^−1^
Subscripts	
0	inlet of the membrane reactor
*i*	component
*L*	outlet of the membrane reactor
